# Milk fatty acid profile from grass feeding strategies on 2 Holstein genotypes: Implications for health and technological properties

**DOI:** 10.3168/jdsc.2022-0273

**Published:** 2023-02-16

**Authors:** N. Techeira, K. Keel, A. Garay, F. Harte, A. Mendoza, A. Cartaya, S. Fariña, T. López-Pedemonte

**Affiliations:** 1 Unidad de Ciencia y Tecnología de Lácteos, Universidad Tecnológica del Uruguay, 70200, La Paz, Colonia, Uruguay; 2 Department of Food Science, Pennsylvania State University, State College 16802; 3 Instituto Nacional de Investigación Agropecuaria (INIA), Programa de Producción de Leche, Estación Experimental INIA La Estanzuela, Ruta 50 km 11, 39173, Colonia, Uruguay

## Abstract

The objective of the study was to determine if a feeding system with a variable supply of grass promoted rapid changes in the fatty acid profile and technological and health indices of milk obtained from North American (NAHF) and New Zealand (NZHF) Holstein-Friesian cows. Two feeding strategies were conducted: fixed grass (GFix) and maximized grass intake when available (GMax). The results showed that as the grass intake increased in the GMax treatments, the relative amount of palmitic acid in milk decreased, whereas oleic, linoleic, linolenic, and conjugated linoleic acids increased, causing a reduction in the atherogenic, thrombogenic, and spreadability calculated indices. The changes occurred in rapid response to the changing diet, with reductions ranging from approximately 5 to 15% in the healthy and technological indices within a period of 15 d of grass intake increase. Differences were found between the 2 genotypes, with NZHF responding faster to changes in grass intake.

## Highlights

• Fatty acid profile in milk changed rapidly with changes in grass intake.
• Health lipids and technological indices changed quickly when grass intake varied.
• Milk showed better nutritional and technological quality when grass feeding increased.
• Changes in some fatty acids due to grass intake were determined by genotype.

## Graphical Abstract

**Figure:**
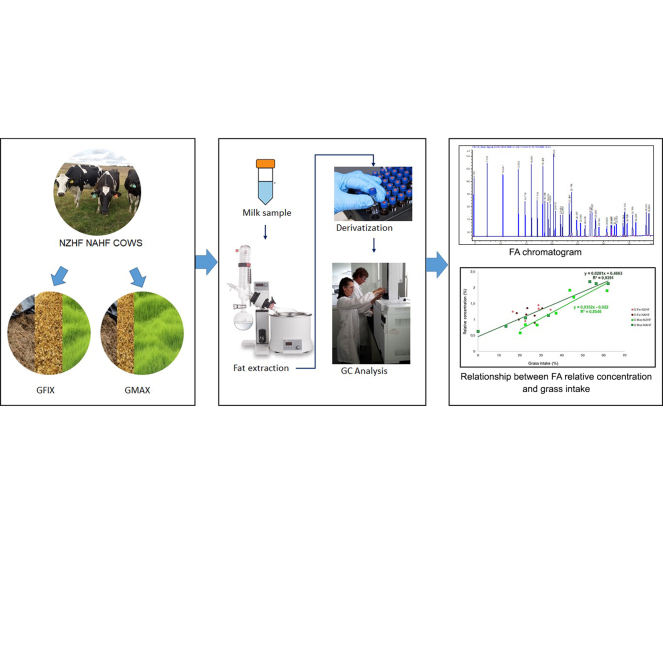
**Summary:** The study evaluated the effect of 2 feeding strategies, fixed grass (GFix) and maximized grass (GMax), on the fatty acid composition of milk obtained from North American (NAHF) and New Zealand (NZHF) Holstein-Friesian cows, determined by gas chromatography of the derivatized milk fat extract. The results showed that there is a significant correlation between grass intake and fatty acid relative concentration, which affects health and technological indices.

Bovine milk typically contains 3–5% fat, composed of approximately 400 different fatty acids (**FA**), represented by 50–70% of SFA, 20–40% of MUFA, and 1–5% of PUFA (Pegolo et al., 2016). Milk and dairy products are sources of functional lipids, including CLA, oleic acid, n-3 FA, short- and medium-chain FA, that are relevant for human health (Moscovici Joubran et al., 2021). Butyric acid is a potent inhibitor of cancer cell proliferation (Ledoux et al., 2005); consumption of CLA has anticarcinogenic, antidiabetic, and antiatherogenic effects (Khanal et al., 2008) and is inversely correlated with obesity (Bär et al., 2020).

The FA profile of milk also determines its technological properties. The levels of SFA and UFA govern the texture and hardness of butter. Butters made with cream rich in UFA are softer and more spreadable than butters made with cream rich in long-chain SFA (O'Callaghan et al., 2016; Marangoni and Ghazani, 2021).

In ruminants, milk FA originate from preformed sources (diet and mobilized adipose tissue) and from de novo synthesis in the mammary gland. Short- and medium-chain FA (≤14 carbons) and approximately 50% of 16-carbon FA are the end products of de novo synthesis. The rest of 16-carbon and longer chain FA are captured from the blood circulation and originate mainly from the diet and through ruminal biohydrogenation processes. Linoleic and linolenic FA cannot be synthesized and their presence in milk depends on the diet; however, through ruminal biohydrogenation they can form intermediate metabolites such as vaccenic acid (**VA**) and CLA (Tanaka, 2005).

The FA composition of cow milk is affected by intrinsic factors (breed, genotype, parity, stage of lactation, and milk yield) and extrinsic factors (diet composition, herd management, milking frequency, and season; Samková and Kalác, 2021).

Several studies demonstrated that including more grass in the diet results in higher fat content in milk and increased proportion of MUFA, PUFA, and CLA (White et al., 2001). Stergiadis et al. (2019) concluded that a higher intake of fresh grass, a higher proportion of forage:concentrate ratio, and the supplementation with plant oils, oilseeds, or protected lipids with an elevated content of UFA, decrease SFA and increase PUFA, n-3, and rumenic acid. Similarly, Couvreur et al. (2006) and O'Callaghan et al. (2016) demonstrated that higher fresh grass intake caused a linear increase in UFA, which translates in a modification in the butter spreadability index (**SI**; C16:0/C18:1), decreasing final melting temperature and reducing perceived mouth firmness.

Additionally, the increase in UFA causes the reduction of the atherogenic (**ATI**) and thrombogenic (**TI**) indices, improving the nutritional value of butters. The ATI indicates the tendency of FA to adhere to cells of the circulatory and immunological systems, whereas TI indicates the tendency to form clots in blood vessels (Chen and Liu, 2020).

Considerable numbers of milk production systems worldwide are based on confined environments (e.g., free stall), with high levels of concentrate supplementation. In these systems, North American Holstein-Friesian (**NAHF**) cows were selected for increased milk yield. In contrast, New Zealand Holstein-Friesian (**NZHF**) cows were selected for increased milk solids production and efficiency under grass feeding systems (O'Sullivan et al., 2019).

The aim of the present study was to evaluate the effect of 2 contrasting feeding systems in 2 Holstein genotypes (NAHF and NZHF) on the milk FA profile and FA-based health and butter technological indices. Emphasis was placed on determining if a feeding system with a variable supply of grass produced quick changes in the fatty acid profile over time, and in the technological and health indices of milk.

The experiment was conducted at the experimental station of the Instituto Nacional de Investigación Agropecuaria (INIA, 34°20′S, 57°41′W, Colonia, Uruguay), over a 4-mo period. Animal care and handling procedures were carried out in accordance with the research protocols involving animals approved by the INIA Bioethics Committee of Animal Experimentation (protocol no. 2017.2).

A completely randomized block design with a 2 × 2 factorial arrangement of treatments resulted from the combination of 2 genotypes: NZHF or NAHF, and 2 feeding strategies, according to the proportion of grazed pasture in the annual feeding budget: fixed grass feeding (**GFix**) and maximized grass feeding (**GMax**). The general design, feeding system, amounts of grass intake over time, and animal management are described in detail in Stirling et al. (2021); the study corresponded to the winter to spring season in yr 1. Briefly, GFix consisted of a feeding strategy with a 1:1:1 ratio of grass, concentrate, and corn and grass silage. The GMax strategy was designed to maximize the proportion of grass in the overall diet, with a flexible grass allowance per cow and day, which was determined by dividing the weekly grass growth rate by the weekly stocking rate; concentrate was fixed to approximately 33% of the diet, and corn and grass silage was used as a buffer when the grass supply decreased. Actual grass intake was determined by the difference between pre- and postgrazing herbage mass (as described by Stirling et al., 2021).

Cows were randomly assigned to each treatment before calving, ensuring that groups were balanced for expected calving date, parity, yield, and milk components from the previous lactation (Stirling et al., 2021). Each treatment (GFix-NZHF, GFix-NAHF, GMax-NZHF, and GMax-NAHF) had 30 cows. Milk samples were obtained from a subsample of 10 cows randomly selected from a pool of cows having similar DIM, parity, and daily milk production (within each genotype) to reduce bias and experimental error.

At the beginning of the sampling period, NZHF cows had a BCS of 2.49 ± 0.19, 89.87 ± 29.83 DIM, parity of 2.31 ± 1.23, and 25.5 ± 4.17 L of daily milk production; NAHF cows had a BCS of 2.45 ± 0.20, 95.22 ± 29.97 DIM, parity of 2.51 ± 1.21, and 30.01 ± 5.40 L of daily milk production.

Samples of milk were collected every 15 d, for a total of 7 samples per cow in 4 mo. Fifty percent of the milk from each sample corresponded to the morning milking and the remaining 50% to the afternoon milking. Since each of the 4 treatments consisted of 10 cows, the total number of samples obtained for analyses was 280.

During the first 3 samplings, average pasture intake (expressed as a percentage of total DMI) was 12.0 ± 8.0, 23.8 ± 3.0, 24.2 ± 2.8, and 26.4 ± 2.8 for GMax-NAHF, GMax-NZHF, GFix-NAHF, and GFix-NZHF, respectively, and average total intake (expressed as kg of DM/cow per d) was 21 ± 2, 20 ± 4, 21 ± 3, and 17 ± 2 for GMax-NAHF, GMax-NZHF, GFix-NAHF, and GFix-NZHF, respectively. During the last 4 samplings, average pasture intake (expressed as a percentage of total DMI) was 51 ± 8, 47 ± 7, 26 ± 4, and 28 ± 7 for GMax-NAHF, GMax-NZHF, GFix-NAHF, and GFix-NZHF, respectively, and average total intake (expressed as kg of DM/cow per d) was 22 ± 3, 18 ± 2, 21 ± 3, and 18 ± 3 for GMax-NAHF, GMax-NZHF, GFix-NAHF, and GFix-NZHF, respectively. The grass proportion in the diet varied from 17 to 35% for GFix-NZHF, from 19 to 31% for GFix-NAHF, from 20 to 62% for GMax-NZHF, and from 0 to 62% for GMax-NAHF, considering that the cows were under a flexible grass allowance.

Milk fat of collected samples was extracted according to Röse-Gottlieb reference method [ISO 1211:2010 (E), IDF 1:2010 (E); ISO, 2010]. Once the milk fat from each sample was extracted, the FA were derivatized according to the method ISO 15884:2002–IDF 182:2002 (ISO, 2002) for the preparation of FAME.

The derivatized samples were analyzed using a GC (model 7820a, Agilent Technology) fitted with a DB-FFAP column (30 m × 0.250 mm i.d., 0.25 µm film thickness, Agilent Technology), according to Firl et al. (2014) with modifications. The split/splitless injector was used with split 50, and samples were injected at 40°C. First, the oven temperature was maintained for 3 min at 40°C, then raised by 15°C∙min^−1^ to 100°C and maintained for 2 min, raised by 10°C∙min^−1^ to 200°C and maintained for 10 min. Finally, temperature was raised by 5°C∙min^−1^ to 240°C and maintained for 10 min. Injector and detector temperatures were 240°C and 250°C, respectively. Nitrogen was used as carrier gas and as make-up gas, with a constant flow of 25 mL∙min^−1^.

The FA identification was performed considering the retention time of a standard mix of 37 FA (product reference number: PE1207, Sigma-Aldrich) and of a CLA standard, constituted by a mixture of *cis*-9,*trans*-11 and *cis*-10,*trans*-12 octadecadienoic acid methyl esters (product reference number O5632, Sigma-Aldrich). The FA quantification was expressed as percent relative concentration using the area under the curve generated for each peak of FA identified obtained in the chromatogram.

To evaluate the nutritional importance of obtained milk, the ATI and TI were calculated as described by Ulbricht and Southgate (1991), as follows:

$$ATI=\frac{\left[C12:0+\left(4\times C14:0\right)+C16:0\right]}{\Sigma \;UFA},$$

$$\begin{array}{c} TI=\\ \frac{\left(C14:0+C16:0+C18:0\right)}{\left(0.5\times MUFA\right)+\left(0.5\times \;\Sigma n-6\;PUFA\right)+\left[3\times \;\Sigma n-3PUFA+\left(\frac{n-3}{n-6}\right)\right]}, \end{array}$$

where C12:0 is lauric FA, C14:0 is myristic FA, C16:0 is palmitic FA, and C18:0 is oleic FA.

In addition, the technological potential of milks to obtain butter was evaluated using SI (as a ratio of C16:0 to C18:1), proposed by O'Callaghan et al. (2016).

Statistical analysis of the results consisted of a first-order simple linear regression analysis to evaluate the existence of a significant relationship between the proportion of grass ingested and the following response variables: relative concentration of the group of VFA (C4:0, C6:0, C8:0, C10:0), certain SFA (C11:0, C12:0, C13:0, C14:0, C15:0, C16:0, C17:0, C18:0), certain UFA (C14:1, C15:1, C16:1, C18:1, C18:2, C18:3, CLA), ATI, TI, and SI. Coefficients of determination (R^2^) were reported, and *P*-values less than 0.05 were deemed significant.

In addition, to evaluate the response of each genotype to the intake of grass, an ANOVA of the slope of each one of the response variables for individual cow was done to check if there were significant differences between the responses of the genotypes. The software InfoStat was used for statistical analysis (Di Rienzo et al., 2020).

In the case of GFix cows, the linear regression analyzes was not applied because these treatments were designed as control groups, with a constant grass intake. However, the average values obtained in this case for each fatty acid and for the indices were shown in the graphs for comparison.

Significant first-order linear regressions between the proportion of grass intake and most response variables (Figures 1, 2, and 3) were observed for NZHF and NAHF GMax cows. For fat content, VFA, and most medium-chain FA relative concentrations, no significant differences were observed (data not shown).

**Figure 1:**
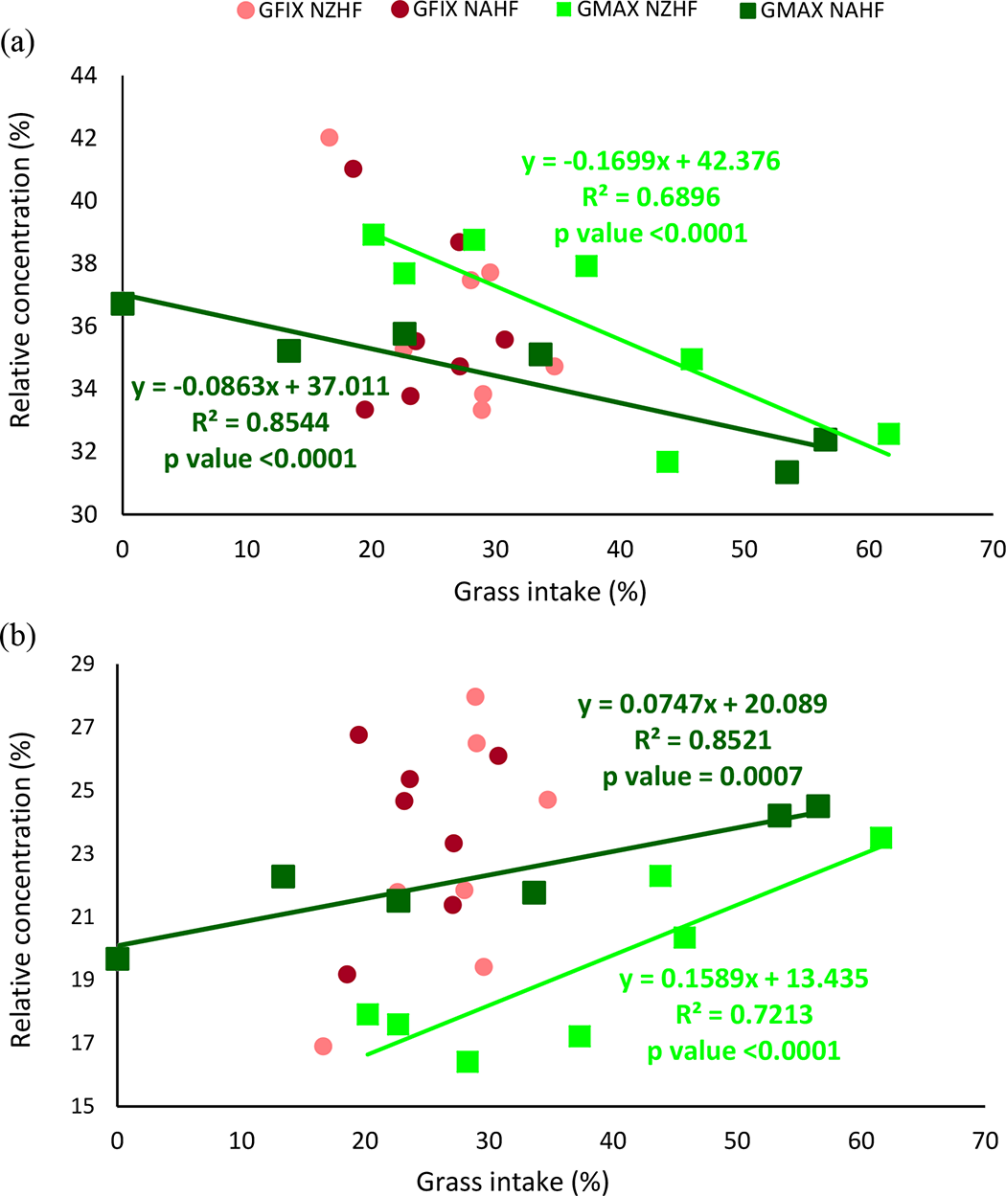
Relationship between grass intake (%) and relative concentration (%) of (a) palmitic and (b) oleic fatty acids present in the milk from cows of fixed grass New Zealand (GFix NZHF), fixed grass North American (GFix NAHF), maximized grass New Zealand (GMax NZHF), and maximized grass North American (GMax NAHF) Holstein-Friesian.

**Figure 2:**
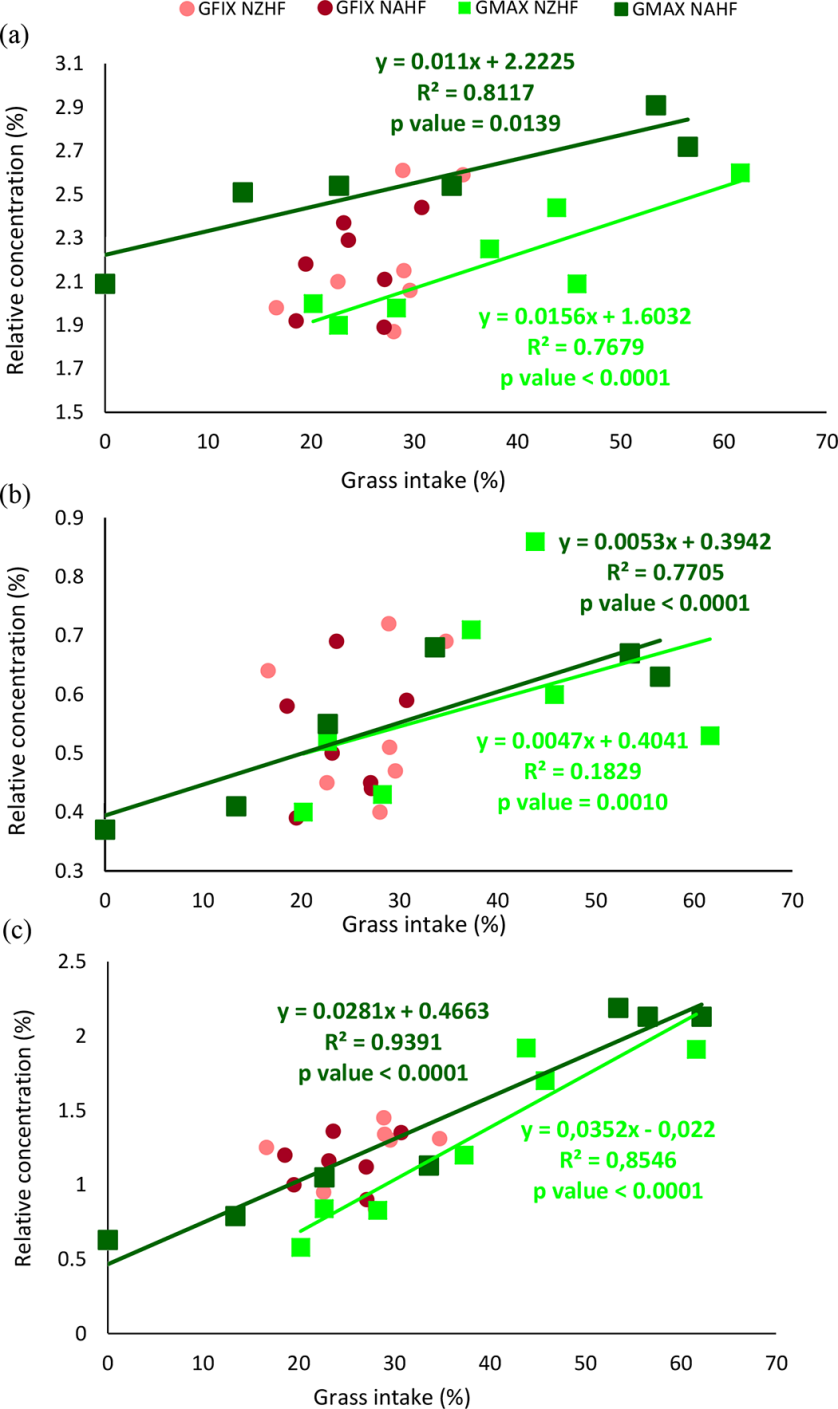
Relationship between grass intake (%) and relative concentration (%) of (a) linoleic, (b) linolenic, and (c) CLA fatty acids present in the milk from cows of fixed grass New Zealand (GFix NZHF), fixed grass North American (GFix NAHF), maximized grass New Zealand (GMax NZHF), and maximized grass North American (GMax NAHF) Holstein-Friesian.

**Figure 3:**
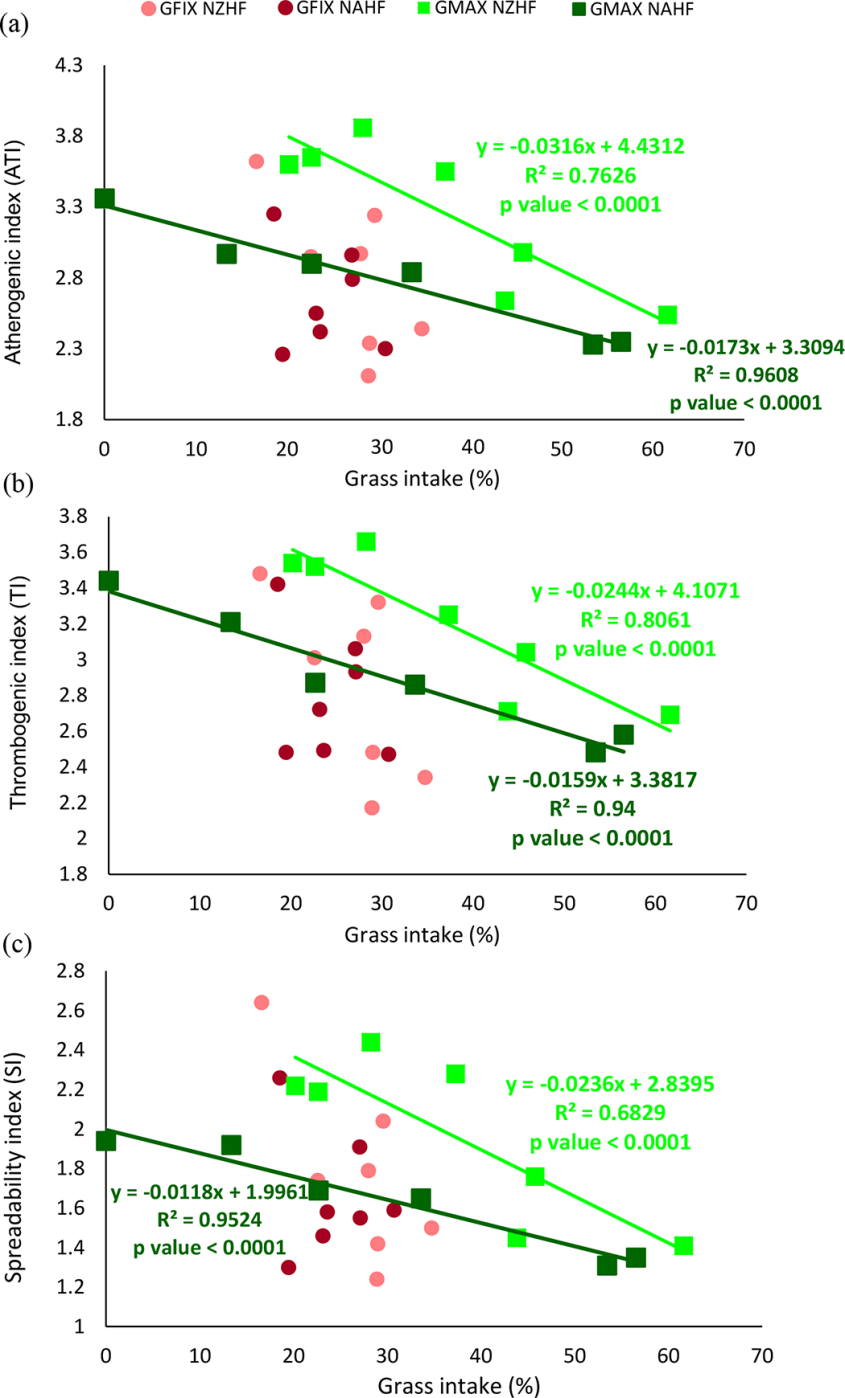
Relationship between grass intake (%) and (a) atherogenic, (b) thrombogenic, and (c) spreadability indices, calculated for the milk from cows of fixed grass New Zealand (GFix NZHF), fixed grass North American (GFix NAHF), maximized grass New Zealand (GMax NZHF), and maximized grass North American (GMax NAHF) Holstein-Friesian.

As the percentage of grass in the diet increased for the GMax treatments, the relative concentration of palmitic FA decreased (Figure 1a), whereas the relative concentration of oleic (Figure 1b), linoleic (Figure 2a), linolenic (Figure 2b), and CLA (Figure 2c) FA increased, in both genotypes. These results were consistent with previous reports where increasing the grass intake in cows of different breeds reduced the concentration of palmitic FA (Couvreur et al., 2006; Capuano et al., 2015; O'Callaghan et al., 2016) and increased all C18 FA (Capuano et al., 2015), MUFA (oleic and vaccenic), and PUFA (linoleic, linolenic and CLA) (Couvreur et al., 2006; Villeneuve et al., 2013).

The increase in the concentration of linoleic and linolenic acids is likely due to a higher grass intake, since these FA cannot be synthesized and their presence in milk only depends on the diet (Glasser et al., 2013).

In addition, biohydrogenation of linoleic and linolenic acids in the rumen increases CLA content in milk. A higher supply of UFA and long-chain FA from grass exceeds the processing capacity by ruminal microbiota and favors the flow of stearic (C18:0) and VA (C18:1 *trans*-11) to the mammary gland, which are then transformed into oleic acid (C18:1 *cis*-9) and CLA, respectively (mainly, by Δ^9^ desaturase enzyme action). Furthermore, the increase of long-chain FA concentration in the mammary tissue causes a further decrease in the de novo synthesis of palmitic acid (Tanaka, 2005; Chamberlain et al., 2014).

No significant effect was found for any genotype between a cow's grass intake and total fat content and relative concentration of VFA (data not shown). This is relevant as the data suggest that the rancidity potential of milk did not change with the diet, with dairy products potentially exhibiting desirable sensory characteristics, regardless of the proportion of grass ingested by the cows. The same behavior was observed for the medium-chain undecanoic (C11:0), lauric (C12:0), tridecanoic (C13:0), myristic (C14:0), myristoleic (C14:1), pentadecanoic (C15:0), palmitoleic (C16:1), heptadecanoic (C17:0), and stearic (C18:0) FA (data not shown).

These results are similar to data reported by Khanal et al. (2008) and by White et al. (2001), who found no differences in the concentration of short- and medium-chain FA in the milk of cows fed exclusively on grass versus a mixed feeding regimen.

However, Couvreur et al. (2006) and Villeneuve et al. (2013) reported that short-chain FA decreased linearly with increased grass intake. These results were consistent with a diet rich in grass that increased the concentration of long-chain FA and PUFA, which in turn inhibited the de novo synthesis of short- and medium-chain fatty acids in the mammary gland. In addition, grazing cows usually ingest less energy than required, which causes substantial reductions in short- and medium-chain FA (Khanal et al., 2008).

As shown in the regression analysis estimation, for every 10% increase in grass intake, the milk from cows on GMax herds dropped the ATI by 0.17 for NAHF cows and 0.31 for NZHF cows (Figure 3a). The TI dropped by 0.16 for NAHF and 0.24 for NZHF (Figure 3b) and SI dropped by 0.12 for NAHF and 0.24 for NZHF (Figure 3c).

The reduction in ATI and TI in the milk of cows with greater grass intake was mostly due to the reduction in the relative concentration of palmitic acid in the milk and to the greater proportion of oleic, linoleic, and CLA FA, since the content of lauric, myristic, and stearic FA in milk did not change.

These results agree with ATI and TI reported for Holstein cows by Couvreur et al. (2006), who founded that ATI decreased 0.22 points for a 30% increase in grass intake and 0.47 points to a 40% increase, with ATI ranging between 2.93 and 2.01. O'Callaghan et al. (2016) evaluated milk for butter production, obtaining TI ​​of 4.51 under a mixed feeding system (grass and corn silage) and a lower TI of 4.06 under a grazing system. The literature reports values from 1.60 to 3.79 for ATI, and from 0.39 to 5.04 for TI for milk from Holstein-Friesian cows (Sharma et al., 2018; Chen and Liu, 2020).

In our study, when the maximum of ingested grass was reached (61.62% for NZHF cows and 62.18% for NAHF cows), the ATI decreased to 2.54 and 2.42 for these genotypes, whereas TI decreased to 2.69 and 2.58, respectively. These values are considered low in milk and dairy products (Sharma et al., 2018; Chen and Liu, 2020), so it could be presumed that the consumption of the milks obtained under this feeding strategy (>60% grass in the diet) would be favorable from a consumer health perspective. However, there are no current recommendations from health organizations regarding ATI or TI thresholds.

The observed SI were also consistent with those reported by Couvreur et al. (2006), who determined that cows fed with 0% grass showed mean SI in milk ​​of 1.41, whereas milk from cows fed with 30, 60, and 100% grass showed SI of 1.21, 1.09, and 0.86, respectively. Similarly, O'Callaghan et al. (2016) found that the SI of milk obtained from cows fed with grass (SI = 1.58) was lower than milk from cows under a mixed feeding regimen (SI = 1.73).

The lower SI observed in milk from GMax cows can be explained by the relative reduction in palmitic acid concentration in milk (melting point 62.9°C) and the relative increase in oleic acid (melting point 13–14°C), since these 2 FA were the most abundant. These modifications can potentially affect the hardness and textural properties of butter, since butters made from cream rich in UFA have a smoother and more spreadable texture versus butter made from cream with a higher proportion of SFA (Chamberlain et al., 2014; Marangoni and Ghazani, 2021).

Focusing on the genotypes of the animals, within herds fed with maximum amount of available grass (GMax), the milk from NAHF cows showed lower average absolute content of palmitic FA, and higher content of oleic, linoleic, linolenic, and CLA versus the milk of NZHF cows. These results translated into lower index ATI, TI, and SI, suggesting that the milk obtained from NAHF cows had better lipid quality, and the potential to produce soft butter with improved spreadability.

It is important to highlight the rapid changes in the relative composition of FA in milk as grass intake increased, and how this modified the nutritional and technological indices. For example, when the average grass intake for the NZHF genotype increased 6.9% in 15 d, the ATI, TI, and SI indices dropped by 10.3, 8.2, and 14.4%, respectively, and when the average grass intake for the NAHF cows increased in 10.4% in 15 d, the indices decreased 5.4%, 4.9%, and 5.2%, respectively.

To evaluate the ability of each genotype to respond to variations in grass intake, an ANOVA of the slopes of grass intake versus relative FA content in milk was carried out. Significant differences (*P* < 0.05) between genotypes for palmitic, oleic, linoleic, and CLA FA, as well as for the ATI and SI indices, were found. The NZHF cows showed a faster response in the values of these variables with the change in the proportion of grass ingested.

This difference between the NAHF and NZHF genotypes was also reported by Wales et al. (2009), who evaluated the milk samples obtained from cows fed by 3 different feeding systems (100% grass; 83% grass with 17% concentrate, and 68% grass with 32% concentrate), and found that NAHF cow milk had a lower palmitic FA content and a higher oleic, linoleic, and CLA FA content versus the NZHF cow milk. Genetic selection applied to NZHF genotype cows tends to increase short-chain FA and decrease long-chain FA in grass-based systems, which could be attributed to the increase in milk solids concentration (Roche et al., 2006; Wales et al., 2009).

In conclusion, our study found that a feeding system characterized by variable grass intake in Holstein-Friesian cows from NAHF and NZHF genotypes generates rapid modifications in the relative concentration of palmitic, oleic, linoleic, linolenic, and CLA FA, which in turn have a significant impact on health and the technological indices of milk. The ATI, TI, and SI indices rapidly decreased with the increase in the grass intake, obtaining milks with better lipid quality and technologically suitable to produce butters with softer textural properties. It would be interesting in the future to carry out similar tests with subsequent production of butter and to evaluate if a trained sensory panel can detect the amount of variations observed in these technological indices. The results demonstrated that there were differences between genotypes, where the ability to respond to changes in grass intake was higher in NZHF cows; however, NAHF cows produced milks with more desirable FA profile and butter spreadability index.
